# Supplementary material for: Phenolic Composition and Antioxidant Properties of Cooked Rice Dyed with Sorghum-Leaf Bio-Colorants
Source: Foods. 2021 Aug 31;10(9):2058. doi: 10.3390/foods10092058 (PMC8465656; doi:10.3390/foods10092058)
Supplement: Supplementary file 1 [file foods-10-02058-s001.zip › foods-1329692-supplementary.pdf]

SUPPLEMENTARY FILE – CHROMATOGRAMS OF PHENOLIC STANDARDS

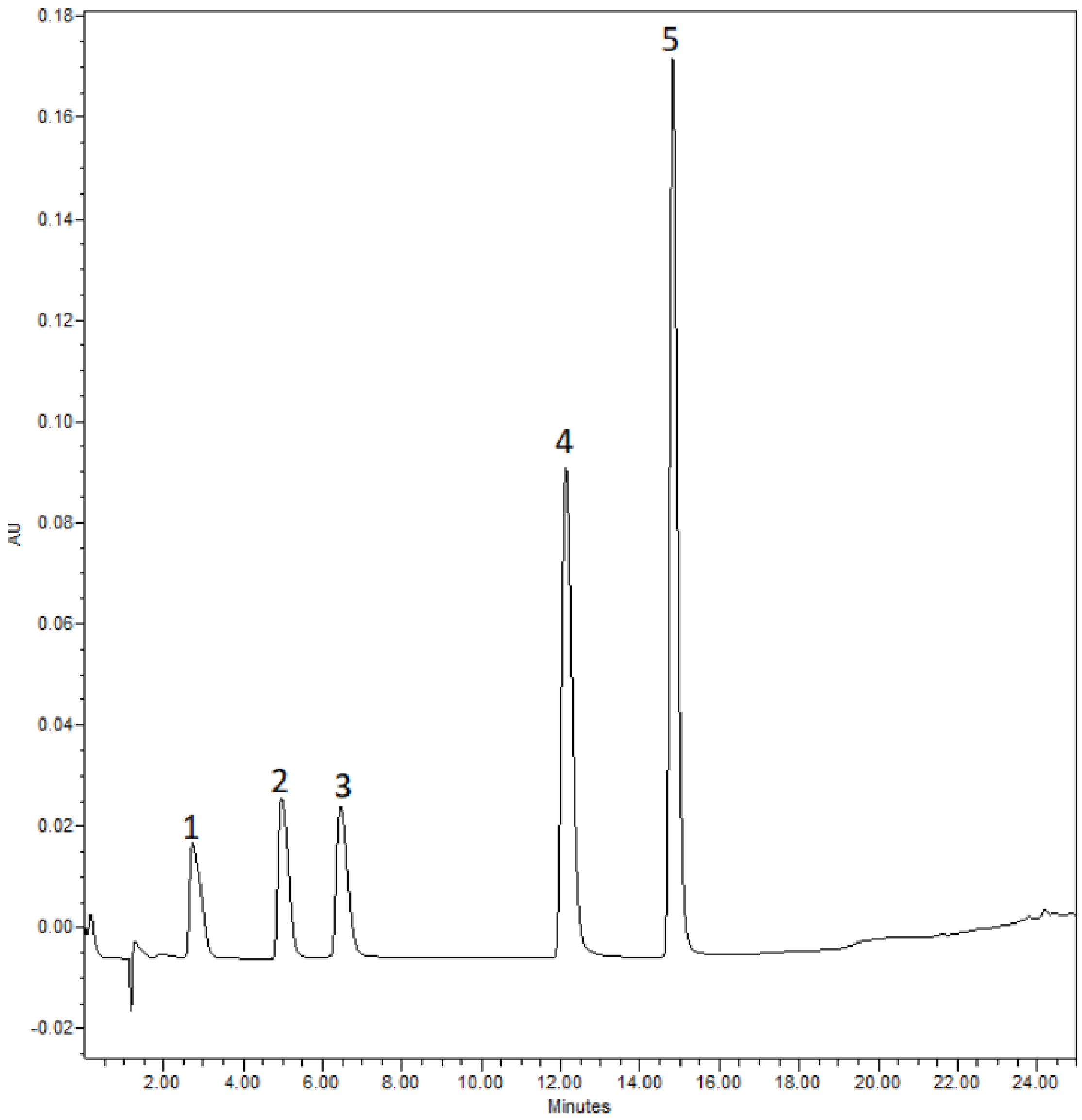

Figure S1. Liquid chromatogram at 280 nm for the HPLC analysis of phenolic acid standards.
Key: 1 = protocatechuic acid (RT = 2.7 min); 2 = *p*-hydroxybenzoic acid (RT = 4.8 min); 3 = 2,4-dihydroxybenzoic acid (RT = 6.3 min); 4 = *p*-coumaric acid (RT = 12.0 min); 5 = ferulic acid (RT = 14.8 min).

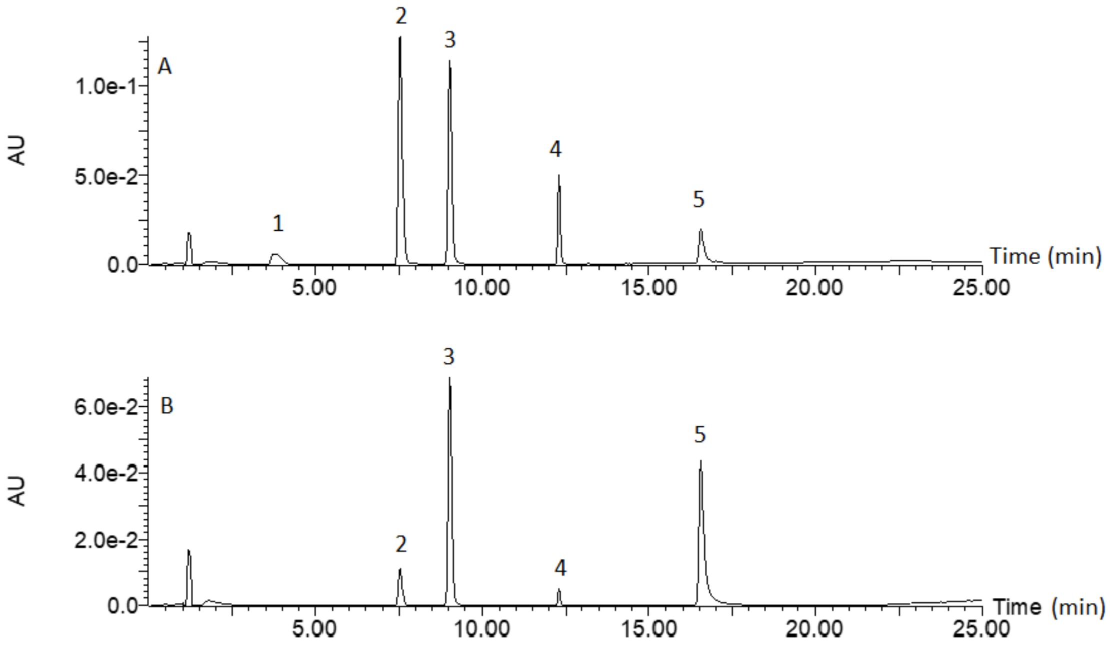

Figure S2. Liquid chromatogram of some phenolic acid and flavonoid standards at 280 nm (A) and 350 nm (B). The phenolic acids were used to quantify coumaric and ferulic acid esters, while the flavonoids were used to quantify the flavonoids and their glycosides.

Key: 1 = (+)-catechin (RT = 3.8 min); 2 = *p*-coumaric acid (RT = 7.5 min); 3 = ferulic acid (RT = 9.0 min); 4 = naringin (RT = 12.3 min); 5 = quercetin (RT = 16.6 min).

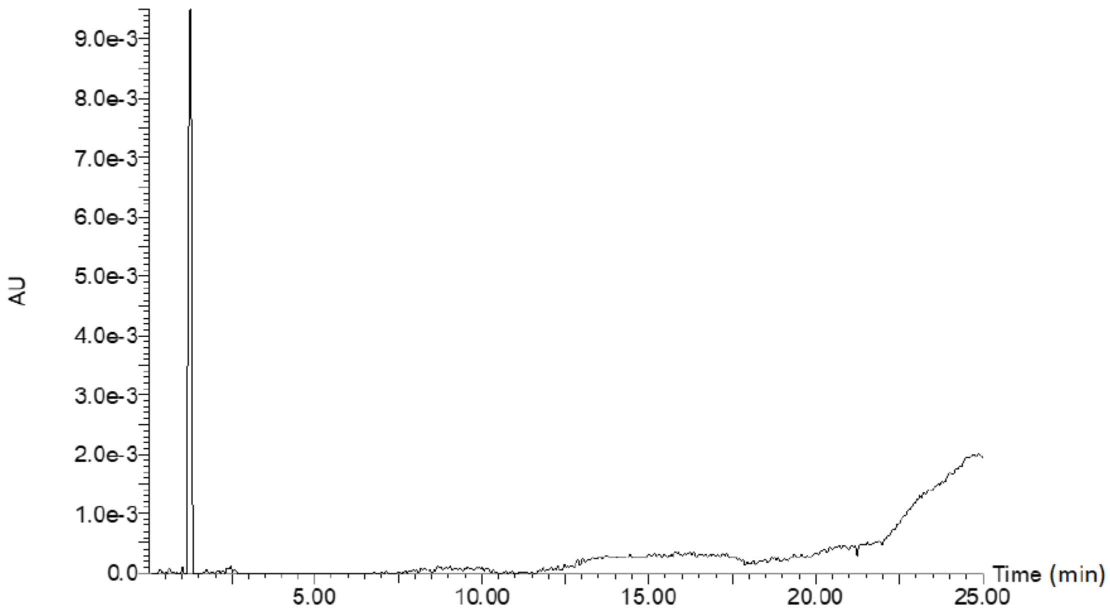

Figure S3. A solvent (baseline) liquid chromatogram for the HPLC analysis of the raw white rice, cowpea, sorghum leaves, their composite blend and cooked products.
